# Clinical implications of bronchoscopy for immune checkpoint inhibitor-related pneumonitis in patients with non-small cell lung cancer

**DOI:** 10.1186/s12890-021-01523-5

**Published:** 2021-05-08

**Authors:** Osamu Nishiyama, Shigeki Shimizu, Koji Haratani, Kosuke Isomoto, Junko Tanizaki, Hidetoshi Hayashi, Ryo Yamazaki, Takashi Oomori, Yusaku Nishikawa, Akiko Sano, Kazuhiko Nakagawa, Yuji Tohda

**Affiliations:** 1 Department of Respiratory Medicine and Allergology, Kindai University Faculty of Medicine, Osakasayama, Osaka Japan; 2 Department of Laboratory Medicine and Pathology, NHO Kinki-Chuo Chest Medical Center, Sakai, Osaka Japan; 3 Department of Medical Oncology, Kindai University Faculty of Medicine, Osakasayama, Osaka Japan

**Keywords:** Alveolitis, Bronchoalveolar lavage, Lung biopsy, Lymphocyte, Organizing pneumonia

## Abstract

**Background:**

The utility of bronchoscopy for patients with suspected immune checkpoint inhibitor (ICI)-related pneumonitis is currently debatable. The purpose of this study was to examine the findings of bronchoalveolar lavage (BAL) analysis and transbronchial lung biopsy (TBLB) in non-small cell lung cancer (NSCLC) patients with ICI-related pneumonitis, and to elucidate the clinical significance of bronchoscopy for this health condition.

**Patients and methods:**

Consecutive NSCLC patients treated with ICIs, diagnosed with ICI-related pneumonitis after undergoing bronchoscopy between October 2015 and March 2019 were retrospectively screened. Findings of BAL fluid analysis and/or TBLB specimen histology were reviewed.

**Results:**

Twelve patients underwent bronchoscopy for the diagnosis of ICI-related pneumonitis, ten of whom underwent BAL. An increase in the proportion of lymphocytes higher than 20% was observed in all ten patients. An increase in the proportion of neutrophils (> 10%) and eosinophils (> 10%) was observed in two and one patient, respectively. TBLB specimens were analyzed for eight patients. Major histologic findings included alveolitis in seven (87.5%) and organizing pneumonia (OP) in five (62.5%) patients. Other findings included acute lung injury and fibrosis. All twelve patients demonstrated favorable outcomes.

**Conclusion:**

A major characteristic of BAL analysis in ICI-related pneumonitis with NSCLC was an increased proportion of lymphocytes. The histologic features of lung tissue included alveolitis and/or OP. Acute lung injury and fibrosis were observed. Although the necessity of bronchoscopy should be determined on a case-by-case basis, it is necessary to assess these parameters when proper differential diagnosis is needed.

## Introduction

Immune checkpoint inhibitors (ICIs) have revolutionized the treatment of many types of cancer. Over the past few years, indications for the use of ICIs have expanded significantly. Although the survival of cancer patients treated with such agents was prolonged, the risk of immune-related adverse events (irAEs), such as dermatitis, colitis, endocrinopathy, hepatotoxicity, neuropathy, and pneumonitis, was increased [1–3]. ICI-related pneumonitis is one of the most common causes of ICI-related deaths [4–6].

In patients with non-small cell lung cancer (NSCLC) who received ICI monotherapy, the incidence of ICI-related pneumonitis for all grades was reported to range from 1 to 12% [7]. Recent meta-analyses reported that the overall incidence of ICI-related pneumonitis is 4.5% [8]. As for severe pneumonitis, the incidence is reportedly 0.8% to 1.5% for grade 3 or higher, and 0.4% for grade 5 [7, 8].

The diagnosis of ICI-related pneumonitis is usually carried out based on clinical signs, including symptomatology, time to onset, and radiological patterns on high-resolution computed tomography (HRCT). Radiographic patterns that are typical of ICI-related pneumonitis include organizing pneumonia (OP), non-specific interstitial pneumonia (NSIP), hypersensitivity pneumonia (HP), diffuse alveolar damage (DAD), acute interstitial pneumonia (AIP), acute respiratory distress syndrome, ground-glass opacity, and non-specific patterns [4, 5, 9]. Diagnosis is straightforward when the clinical picture is consistent with ICI-related pneumonitis. However, it is sometimes difficult when other possible etiologies, such as lymphangitic spread of tumor, infection, alveolar hemorrhage, and acute exacerbation of existing interstitial lung disease are present.

The utility of bronchoscopy for patients with suspected ICI-related pneumonitis is currently debatable. Although some practice guidelines recommend bronchoalveolar lavage (BAL) and/or transbronchial lung biopsy (TBLB) for suspected ICI-related pneumonitis cases of grade 2 and above [1–3], these processes are not generally performed in clinical practice.

The purpose of this study was to examine the findings of BAL and/or TBLB in NSCLC patients with ICI-related pneumonitis, and to elucidate the clinical significance of bronchoscopy in such patients.

## Methods

### Patients

Consecutive patients with NSCLC who had been treated with ICIs at our university hospital, and who had been diagnosed with ICI-related pneumonitis after undergoing bronchoscopy between October 2015 and March 2019 were retrospectively screened. The findings of BAL analysis and/or histology of TBLB specimens were reviewed.

Chest HRCT findings obtained prior to bronchoscopy were also reviewed. Patterns of the HRCT findings were determined according to the classification proposed previously [4, 5, 9].

### BAL and TBLB

Bronchoscopy was performed based on the physician’s decision because formal clinical indications have not been established. However, it was encouraged for all patients with suspected ICI-related pneumonitis during the study period. BAL was performed with four (50 mL each) aliquots of sterile saline solution, followed by manual aspiration using a bronchoscope [10]. The aspirates of four consecutive aliquots were pooled, and centrifuged to separate the fluid from the cells. Cell differentials were obtained by manual enumeration on cytospin preparations using a Diff-QuikTM stain (Scientific Products, McGraw Park, IL). Cell surface markers were examined using fluorescein isothiocyanate-conjugated CD4 and CD8 monoclonal antibodies (Ortho Diagnostics Inc., Raritan, NJ, USA) by flow cytometry.

TBLB specimens were obtained mainly from the lobes in which lung abnormalities were observed. The histological findings were reviewed by a pathologist (S.S.) who specializes in interstitial lung diseases.

### Treatment and response

Treatments performed for ICI-related pneumonitis were reviewed. Treatment responses were also evaluated.

### Statistical analysis

Continuous variables are summarized as mean ± standard deviation (SD), and categorical variables by the actual numbers. Analyses were performed with Statflex ver.7 (Artec Co., Ltd., Osaka, Japan).

## Results

**Fig. 1:**
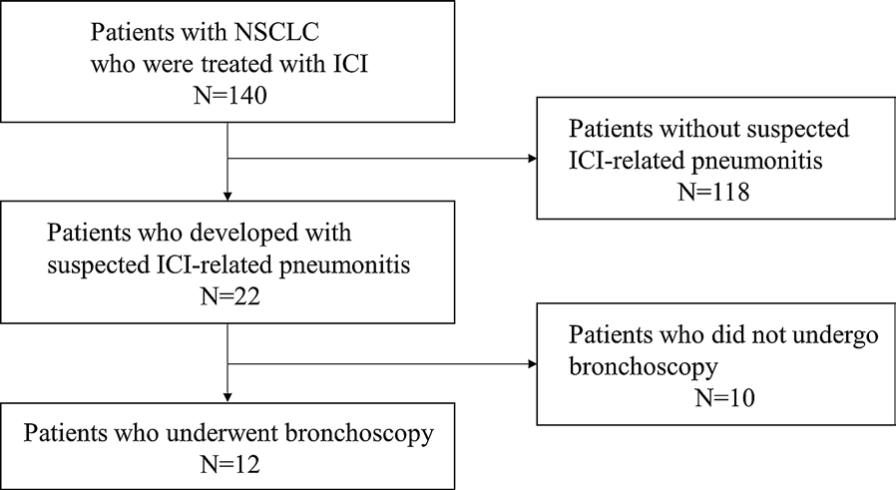
Flowchart depicting the selection of patients for the study

One hundred and forty consecutive patients with NSCLC were treated with ICIs during the study period. Of these, 22 patients were suspected to have ICI-related pneumonitis, and 12 of them underwent bronchoscopy for diagnosis (Fig. 1). Patients who underwent bronchoscopy were admitted to our hospital before the procedure. Subsequently, all 12 patients were clinically diagnosed with ICI-related pneumonitis, and other etiologies were excluded.

Patient characteristics at admission are shown in Table 1. The mean age was 68.8 ± 6.9 years, for nine men and three women. Among the patients, eight had adenocarcinoma, three had squamous cell carcinoma, and one had NSCLC that was not specific. Pre-admission ICI treatment included nivolumab and pembrolizumab for seven and five patients, respectively. The ICIs were used as a single-agent regimen. Duration of ICI use before the diagnosis of ICI-related pneumonitis was 5.0 ± 4.1 month. All patients complained of dyspnea of modified Medical Research Council (mMRC) grade 1 to 4, except three who had no dyspnea. The grade of pneumonitis was 1 in two, 2 in seven, and 3 in three patients; none of the patients had grade 4 pneumonitis.

**Table 1:** Characteristics of the selected patients at admission (n = 12)

Variables	Values
Age, yrs	68.8 ± 6.9
Gender, male/female	9/3
Types of NSCLC, adeno/squamous/NOS	8/3/1
Types of ICI, nivolumab/pembrolizumab	7/5
Duration of ICI use, mo	5.0 ± 4.1
mMRC dyspnea grade, 0/1/2/3/4	3/2/5/1/1
Grade of pneumonitis, 1/2/3/4	2/7/3/0

Values are expressed as mean ± standard deviation or by the actual number

Grade of pneumonitis was assessed according to CTCAE v5.0

CTCAE, Common Terminology Criteria for Adverse Events; ICI, immune checkpoint inhibitor; mMRC, modified Medical Research Council; NSCLC, non-small cell lung cancer; NOS, not otherwise specified

The findings of BAL analysis are presented in Table 2. BAL was performed in ten of the twelve patients who underwent bronchoscopy. Comparing with upper limits of normal for nonsmokers that were proposed by the American Thoracic Society practice guideline (lymphocytes: 15%, neutrophils: 3%, eosinophils: 1%) [10], the proportions of lymphocytes, neutrophils, and eosinophils were increased by 33.5 ± 24.6%, 1.3 ± 5.7%, 2.5 ± 5.6% above the upper limit. In all ten patients the proportion of lymphocytes increased > 20%. The proportion of neutrophils was found to be > 10% in two patients, whereas of > 10% in the proportion of eosinophils was observed in one patient.

**Table 2:** Findings of bronchoalveolar lavage analysis

Case	Age	Gender	Smoking status	Site	Recovery rate (%)	TCC (× 10^5^/mL)	AM (%)	Lym (%)	Neu (%)	Eos (%)	CD4:CD8
1	68	M	Former	–	–	–	–	–	–	–	–
2	73	F	Never	rt. B^10^	49.0	2.1	52.6	44.8	1.8	0.8	1.5
3	68	M	Former	rt. middle lobe	59.5	1.6	47.0	46.4	5.8	0.8	0.3
4	66	M	Former	lt. B^5^	58.0	2.7	66.4	29.0	1.6	3.0	0.5
5	51	M	Current	rt. B^5^	59.0	2.2	5.8	90.4	0.8	3.0	0.5
6	79	M	Former	lt. B^4^	49.0	1.3	12.2	86.0	1.4	0.4	0.9
7	70	M	Former	lt. B^8^	43.5	2.5	20.4	76.6	1.4	1.6	3.6
8	74	M	Former	rt. B^3^	17.5	0.4	32.2	47.8	15.6	4.4	0.5
9	72	M	Former	–	–	–	–	–	–	–	–
10	71	M	Former	rt. B^4^	57.0	3.5	54.0	30.0	14.0	2.0	0.1
11	63	F	Never	rt. B^4^	70.2	2.3	16.2	64.2	0.4	19.2	0.5
12	70	F	Never	rt. B^2^	41.5	1.2	78.8	20.4	0.4	0.4	0.5

Cases 1–5, 7, 8, 12: adenocarcinoma; Cases 6, 9, 11: squamous cell carcinoma; Case 10: not otherwise specified

AM, alveolar macrophage; CD, cluster of differentiation; Eos, eosinophils; Lym, lymphocytes; Neu, neutrophils; TCC, total cell count

The radiographic patterns in chest HRCT, and the major histological findings of the TBLB specimens are shown in Table 3. Chest HRCT of the twelve patients revealed OP, HP, and NSIP patterns in eight (66.6%), three (25.0%), and one (8.3%) patients, respectively. TBLB specimens were obtained from eight patients. The main histologic findings included alveolitis (mild and moderate thickening of alveolar septa by the infiltration of lymphocytes and plasma cells) and OP (airspace filling by fibroblast plugs). Alveolitis was observed in seven (87.5%), and OP was observed in five (62.5%) out of eight patients whose lung specimens could be obtained; co-occurrence of alveolitis and OP was observed in four (50.0%) patients. Other findings included acute lung injury and fibrosis, which were observed in different patients.

**Table 3:** Radiographic patterns in chest HRCT and histologic findings of transbronchial lung biopsy

Case	Age	Gender	Radiographic patterns in chest HRCT	Major histological findings
1	68	M	OP pattern	Alveolitis, organizing pneumonia
2	73	F	OP pattern	Alveolitis
3	68	M	HP pattern	–
4	66	M	OP pattern	Alveolitis, acute lung injury
5	51	M	HP pattern	–
6	79	M	OP pattern	–
7	70	M	OP pattern	Alveolitis
8	74	M	OP pattern	–
9	72	M	OP pattern	Alveolitis, organizing pneumonia
10	71	M	NSIP pattern	Organizing pneumonia, fibrosis
11	63	F	HP pattern	Alveolitis, organizing pneumonia
12	70	F	OP pattern	Alveolitis, organizing pneumonia

Cases 1–5, 7, 8, 12: adenocarcinoma; Cases 6, 9, 11: squamous cell carcinoma; Case 10: not otherwise specified

The findings of acute lung injury include swelling of type 2 alveolar pneumocytes and detached epithelial cells

HP, hypersensitive pneumonitis; HRCT, high-resolution computed tomography; NSIP, non-specific interstitial pneumonia; OP, organizing pneumonia

**Fig. 2:**
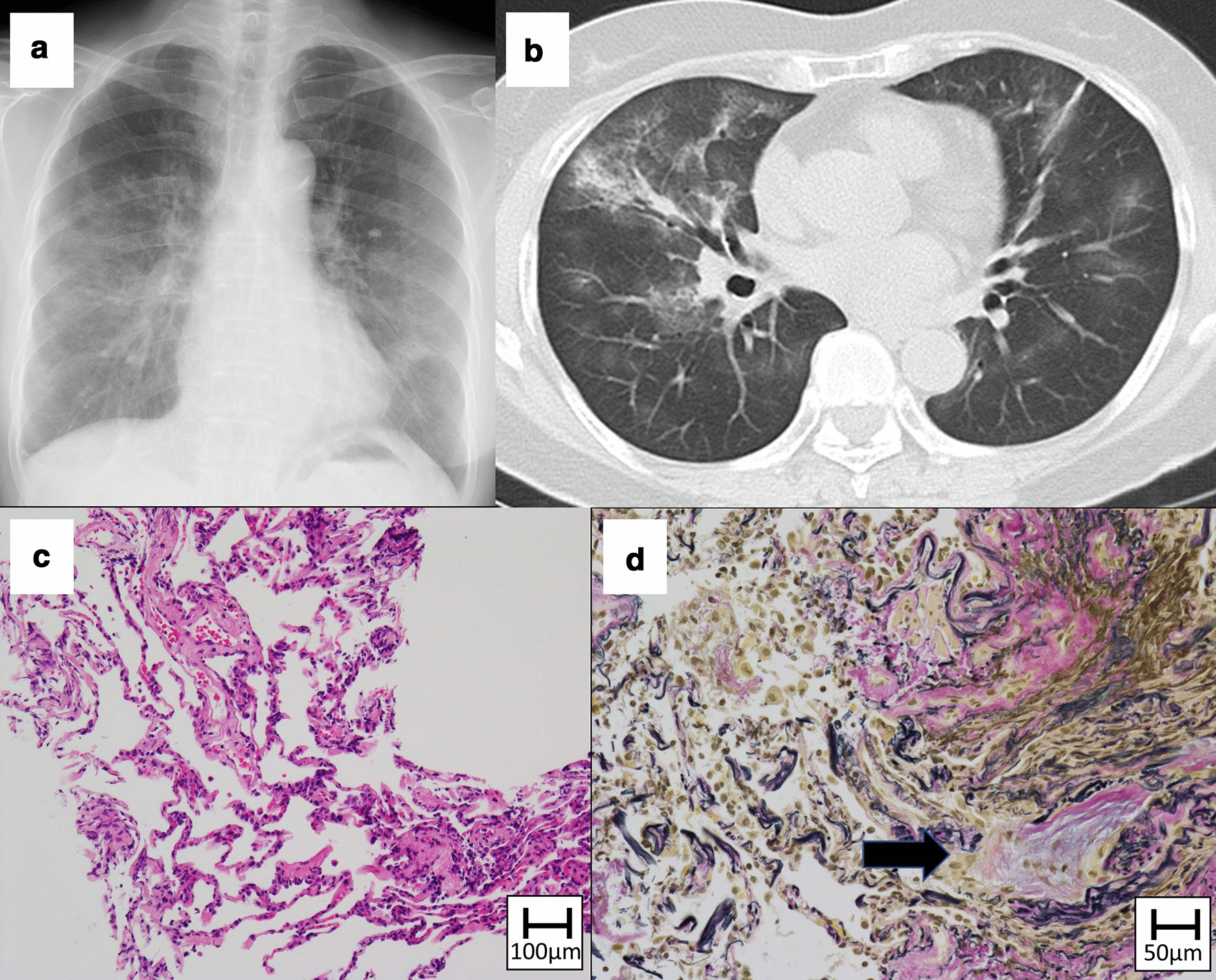
Chest X-ray image showing bilateral lung infiltrates and ground-glass opacities (**A**). High-resolution computed tomography image of chest image showing bilateral pulmonary ground-glass opacities and focal consolidations, suggestive of organizing pneumonia (OP) pattern (**B**). Transbronchial lung biopsy specimen of right S4 shows alveolitis (**C** Hematoxylin and Eosin) and intraluminal OP (arrow) (**D** Elastica van Gieson). (Case No.1)

**Fig. 3:**
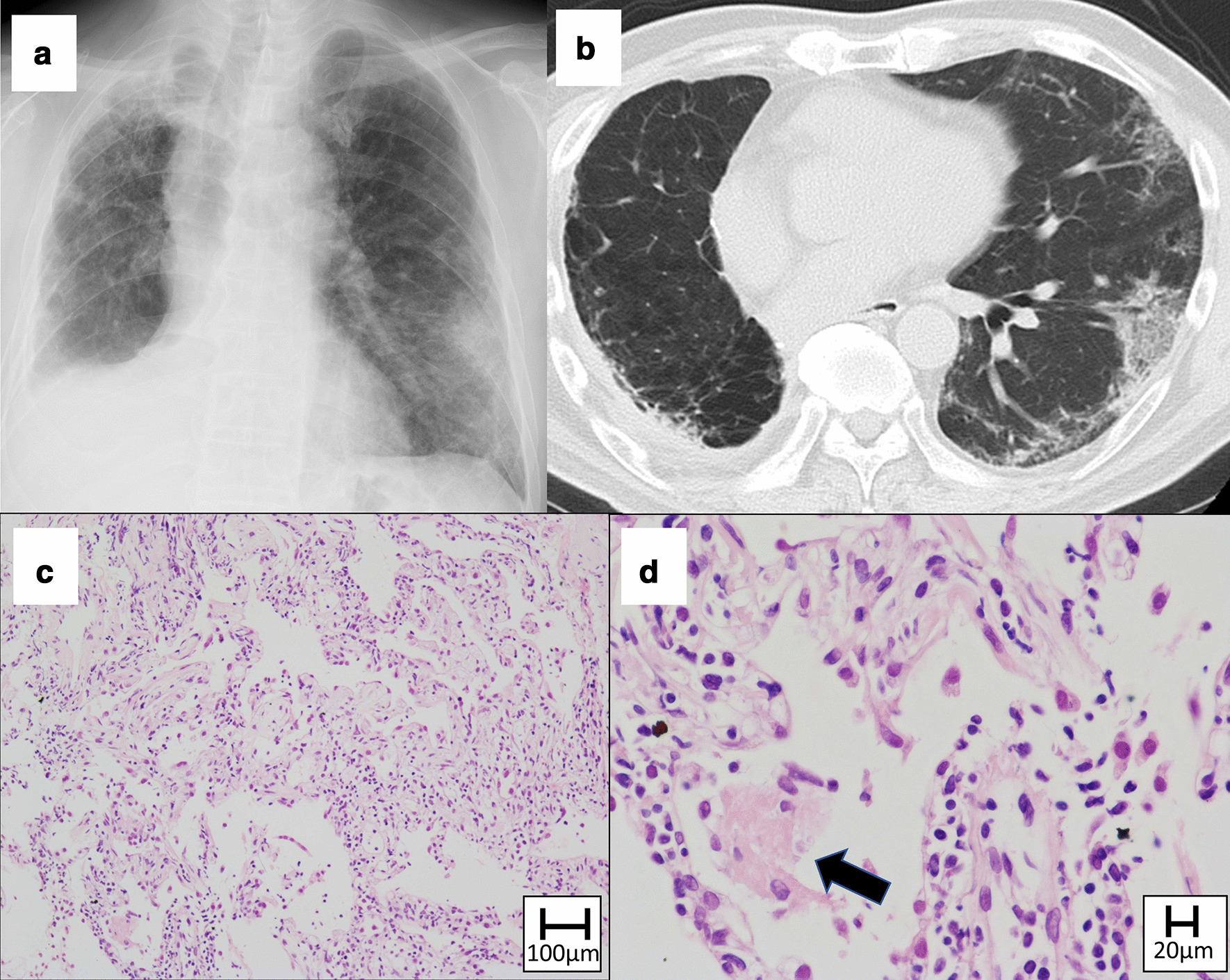
Chest X-ray image showing lung infiltrates in right upper and left middle lung (**A**). High-resolution computed tomography image of chest image showing pulmonary ground-glass opacities in the left upper and lower lobes (**B**). Consolidation is also seen in the right upper lobe, suggestive of organizing pneumonia (OP) pattern. Transbronchial lung biopsy specimen of left S8 shows edema and fibroblast proliferation in alveolar walls, alveolitis (**C** Hematoxylin and Eosin), fibrinous exudate (arrow) in alveolar spaces, and enlarged type 2 pneumocytes (**D** Hematoxylin and Eosin). No hyaline membrane is seen in the specimen. (Case No. 4)

Cases with demonstrative findings of chest radiography, chest HRCT, and TBLB histology are shown in Figs. 2 and 3. Radiographic findings of OP pattern, and histological findings of alveolitis and OP are shown in Fig. 2. Radiographic findings of OP pattern and histological findings of acute lung injury patterns are shown in Fig. 3. The histological findings of acute lung injury pattern revealed edema and fibroblast proliferation in alveolar walls, alveolitis, fibrinous exudate in alveolar spaces, and enlarged type 2 pneumocytes; no hyaline membrane was seen in the specimens. The findings could not be easily classified as OP or diffuse alveolar damage.

Initial treatments for ICI-related pneumonitis are shown in Table 4. Ten of the twelve patients (83.3%) underwent treatment for ICI-related pneumonitis. Initial corticosteroid therapy using 0.5 and 1 mg/kg prednisolone was undertaken for two and six of the patients, respectively. One patient underwent high-dose methylprednisolone therapy (0.5 g/day for three consecutive days). The corticosteroid dose was gradually decreased in all patients treated with corticosteroids only. One patient received 1 mg/kg of prednisolone, followed by infliximab, because the initial response to prednisolone was insufficient. One patient received infliximab therapy only. One patient did not undergo any therapy. Abnormal shadows on chest HRCTs due to ICI-related pneumonitis disappeared in six patients, and improved in the other six after the treatments (Table 4).

**Table 4:** Treatment approach for ICI-related pneumonitis and reactivity for chest HRCT findings

Case	Initial treatment	Chest HRCT findings
1	0.5 mg/kg prednisolone (p.o.)	Disappeared
2	1 mg/kg prednisolone (p.o.)	Disappeared
3	0.5 g methylprednisolone (i.v.) × 3 days followed by 1 mg/kg prednisolone (p.o.)	Markedly improved
4	3 mg/kg infliximab (i.v.)	Markedly improved
5	1 mg/kg prednisolone (p.o.)	Markedly improved
6	0.5 mg/kg prednisolone (p.o.)	Markedly improved
7	1 mg/kg prednisolone (p.o.) and 3 mg/kg infliximab (i.v.)	Disappeared
8	1 mg/kg prednisolone (p.o.)	Markedly improved
9	1 mg/kg prednisolone (p.o.)	Markedly improved
10	1 mg/kg prednisolone (p.o.)	Disappeared
11	1 mg/kg prednisolone (p.o.)	Disappeared
12	none	Disappeared

Daily dose is as shown for prednisolone and methylprednisolone. The dose of prednisolone was gradually decreased in all patients who underwent corticosteroid treatment. Infliximab was administered with a single dose of 3 mg/kg

HRCT, high-resolution computed tomography; ICI, immune checkpoint inhibitor; i.v., intravenous; p.o., per os (oral administration)

The ten patients who did not undergo bronchoscopy consisted of seven with adenocarcinoma, two with squamous cell carcinoma, and one with NSCLC that was non-specific. The mean age of the ten patients was 64.8 ± 15.8 years, which included six men and four women. ICI treatment at admission included nivolumab and pembrolizumab for seven and three patients, respectively; the ICIs were used as a single-agent regimen. The grade of pneumonitis at admission was 1 in three patients, 2 in four, 3 in one, and 4 in two patients. Corticosteroid treatment for ICI-related pneumonitis was provided to nine of the ten patients, whereas one did not receive any treatment. Abnormal shadows on chest HRCTs due to ICI-related pneumonitis improved or disappeared in eight patients. However, two patients died of respiratory failure 3 and 31 days after admission, respectively, despite high-dose corticosteroid therapy; both patients suffered from grade 4 pneumonitis at admission.

## Discussion

In this study, we examined the findings of BAL and/or TBLB in twelve consecutive NSCLC patients with ICI-related pneumonitis. Analysis of BAL indicated of > 20% in the proportion of lymphocytes in all patients who underwent BAL examination. The main histologic features in lung tissue revealed by TBLB included alveolitis and/or OP. Acute lung injury and fibrosis were also observed. All patients who were treated with corticosteroids and/or infliximab showed favorable responses to the treatments; one patient improved without therapy.

ICIs are one of the most important drugs for treating patients with advanced or recurrent NSCLC. Cytotoxic T-lymphocyte-associated protein 4 and programmed death 1 (PD-1)/programmed death ligand 1 (PD-L1) are the most relevant targets for immunotherapy [11]. For patients with NSCLC, ICIs directed at PD-1 (nivolumab and pembrolizumab) were approved first for clinical use in Japan. This was followed by the approval of ICIs directed at PD-L1 (atezolizumab and durvalumab). These ICIs are currently used in clinical practice according to established evidence for each ICI, for example, as first-line treatment, second-line or later treatments, and in combination with cytotoxic chemotherapy [12]. However, as the opportunities for using ICIs increase, the frequency of irAEs is also increasing. In irAEs, pneumonitis is considered important because it can be lethal [4–6].

The pathophysiology of ICI-related pneumonitis is poorly understood. Although some practice guidelines recommend BAL and/or TBLB for suspected ICI-related pneumonitis of grade 2 or higher [1–3], it seems that many patients with clinically suspected ICI-related pneumonitis never undergo bronchoscopy in clinical practice. Although several case presentations have been reported in which findings of BAL and/or TBLB were available, only a few reported the findings in a case series [6, 13]. To our knowledge, the present study is the first to systemically detail BAL and histological findings, and correlate these findings with radiographic findings in NSCLC patients with ICI-related pneumonitis. Notably, pathologic OP was observed not only in patients with radiographic OP patterns, but also in those with HP and NSIP patterns. Pathologic findings suggestive of acute lung injury were observed in patients with radiological OP patterns. The present study suggests that alveolitis and OP are major pathologic findings of lung specimens for patients with ICI-related pneumonitis, which do not necessarily correspond to radiologic patterns.

A previous study demonstrated that the proportion of lymphocytes of > 15% in BAL fluid was observed in 24 out of 30 (80%) patients with ICI-related pneumonitis whose BAL findings could be evaluated, although patients with lung cancer as well as melanoma and others were included [6]. These results were similar to ours. Another study on lung tissues of patients with different types of cancer and ICI-related pneumonitis revealed OP to be a major pathologic finding in seven out of nine (77.8%) patients [13]. Diffuse alveolar damage and acute fibrinous pneumonitis were also observed in one patient each. These findings are also similar to our results. However, it is noteworthy that pathologic findings suggestive of acute lung injury were also seen in a patient in whom the proportion of lymphocytes in BAL fluid increased in the present study. It should be noted that the term acute lung injury pattern is sometimes used by pathologists when both diffuse alveolar damage and OP are present in the same biopsy, or if the findings cannot be easily pigeonholed into either category [14]. Our case with pathologic findings suggestive of acute lung injury was presumably corresponded to this pattern.

Considering the responses to treatments for ICI-related pneumonitis, all patients who underwent bronchoscopy showed favorable clinical courses in the present study. Given that patients with cryptogenic OP of idiopathic interstitial pneumonia usually have a good response to corticosteroids [15], the observation is quite reasonable. None of the patients, including the one whose lung specimen showed pathological findings suggestive of acute lung injury, died of ICI-related pneumonitis. This suggests that the increased proportion of lymphocytes in BAL fluid can be a marker for favorable treatment response, even in patients with lungs showing acute lung injury. Pathological findings of acute lung injury are usually considered refractory to treatment, and have a poor prognosis. This is presumed from the knowledge of idiopathic interstitial pneumonia [15], as well as from the experience of the induction of lung disease by cytotoxic drugs, such as amiodarone, bleomycin, and epidermal growth factor receptor-tyrosine kinase inhibitor. Furthermore, an increased proportion of neutrophils in BAL fluid is usually recognized as a marker of poor prognosis. In the present study, two patients showed increased neutrophil counts in BAL fluid, which was accompanied by an increased lymphocyte count. No patient showed increased neutrophil count in BAL fluid as a major finding.

If bronchoscopy could be performed in patients who died of ICI-related pneumonitis, different findings in the BAL analysis might have been obtained. Given that an increased proportion of neutrophils in BAL fluid was reported in patients with AIP and acute exacerbation of idiopathic pulmonary fibrosis, whose lungs were assumed to have DAD pathologically [16–22], lung specimens from patients with ICI-related pneumonitis and radiological DAD pattern on chest HRCT may show similar patterns in BAL analysis. Further studies are needed to clarify the characteristics of BAL analysis in patients with ICI-related pneumonitis and radiologic DAD patterns in chest HRCT, who would die in spite of an appropriate treatment.

The utility of bronchoscopy in obtaining BAL fluid and lung specimens is still undetermined for patients with suspected ICI-related pneumonitis. However, when a differential diagnosis is crucial for other diseases, such as lung infections including pneumocystis pneumonia, lung edema, and pulmonary hemorrhage caused by anticoagulants and/or antiplatelet drugs, evaluation of BAL fluid and/or pathological findings are considered to be useful. When other diagnoses are excluded and ICI-related pneumonitis is most strongly suggested, an increased proportion of lymphocytes may predict a good response to treatment, and a favorable prognosis. Although indications for bronchoscopy should be determined on a case-by-case basis, discussion between the oncologist in charge of the anticancer therapy and the pulmonologist is important.

There are some limitations to this study. First, our study was performed at a single center, and had a relatively small sample size. Second, this was a retrospective study. Ten of the twenty-two patients did not undergo bronchoscopy. In addition, we could not evaluate BAL and/or TBLB in patients with severe ICI-related pneumonitis. Therefore, markers indicating poor treatment response and prognosis were not evaluated. These limitations warrant further prospective studies including more patients undergoing bronchioscopic evaluation. A similar analysis in patients with ICI-related pneumonitis and other types of cancer should also be performed.

## Conclusions

The major characteristic of BAL analysis in patients with NSCLC and ICI-related pneumonitis was an increase in the proportion of lymphocytes to > 20%. Histologic features of lung tissue obtained by TBLB included alveolitis and/or OP. Acute lung injury and fibrosis were also observed. As all the patients who underwent bronchoscopy demonstrated favorable courses in the study, the increased proportion of lymphocytes in BAL fluid may be an indicator of good treatment response. Although the necessity of bronchoscopy should be determined on a case-by-case basis, it is necessary to assess these parameters discussed in the present study when proper differential diagnosis is needed.

## Data Availability

The datasets used and/or analyzed during the current study are available from the corresponding author upon reasonable request.

## Abbreviations

AIP: Acute interstitial pneumonia
BAL: Bronchoalveolar lavage
DAD: Diffuse alveolar damage
HP: Hypersensitivity pneumonia
HRCT: High-resolution computed tomography
ICI: Immune checkpoint inhibitor
irAE: Immune-related adverse events
mMRC: Modified Medical Research Council
NSCLC: Non-small cell lung cancer
NSIP: Non-specific interstitial pneumonia
OP: Organizing pneumonia
PD-1: Programmed death 1
PD-L1: Programmed death ligand 1
TBLB: Transbronchial lung biopsy
